# Social Enterprise as a Model to Improve Live Release and Euthanasia Rates in Animal Shelters

**DOI:** 10.3389/fvets.2021.654572

**Published:** 2021-04-20

**Authors:** Jennifer Thomsen, Bastian Thomsen, Kellen Copeland, Sarah Coose, Sean Blackwell, Vitoria Dante

**Affiliations:** ^1^Multispecies Livelihoods Lab, Department of Management, College of Business and Economics, Boise State University, Boise, ID, United States; ^2^School of Anthropology and Museum Ethnography, Kellogg College, Institue of Social and Cultural Anthropology, University of Oxford, Oxford, United Kingdom; ^3^School of Business and Law, Central Queensland University, Brisbane, QLD, Australia; ^4^Environmental Studies Graduate Program, Graduate School, Oregon State University, Corvallis, OR, United States; ^5^Anthropology and Sociology Department, College of Idaho, Caldwell, ID, United States; ^6^Department of Human Dimensions of Natural Resources, Warner College of Natural Resources, Colorado State University, Fort Collins, CO, United States

**Keywords:** live release rate, euthanasia, animal shelters, social entrepreneurship, multispecies livelihoods, animal welfare, non-profit organizations/sector, human-animal studies

## Abstract

This paper explored the role that social entrepreneurship may play in helping to improve euthanasia and live release rates in animal shelters. This paper used a qualitative, comparative ethnographic study that included semi-structured interviews, participant observation, and archival research. It compared two large animal shelters from the U.S. and Australia. Collectively, 21 formal interviews, more than 30 informal interviews, and participant observation were conducted over a 6-month time frame between the two countries. Findings indicate that three main factors may contribute to the transformation of non-profit animals shelters and result in improved euthanasia and live release rates, as well as animal caregiver burnout. These include: (1) professionalizing shelter management, (2) engaging with non-profit social enterprise activities, and (3) improving the efficiency of daily operations. In this paper, we argue that by embracing non-profit social enterprise activities, animal shelters may improve anthropocentric animal shelter activities to positively affect human and non-human rights, welfare, and agency. We do not contend that non-profit animal shelters should sacrifice their stated mission or ethics to include business practices. Rather, by professionalizing management and operations that include self-sustaining diverse revenue streams, it may free up time and resources to make a greater effect in positive non-human animal welfare and outcomes.

## Introduction

Euthanasia rates of healthy adoptable dogs (*Canis lupus familiaris*) and cats (*Felis catus*) in the U.S. remain staggeringly high, with ~670,000 dogs and 860,000 cats destroyed annually (1). However, over the past decade, an encouraging trend in companion animal Live Release Rate (LRR) statistics transpired in the United States and Australia (1, 2). “The Asilomar Accords defines LRR as the proportion of animals leaving the shelter alive among those that experience an outcome” [(3), p. 120]. The number of dogs and cats entering U.S. animal shelters each year significantly declined over the last decade, with ~6.5 million dogs and cats entering U.S. shelters in 2019, down 9.7% from an average of 7.2 million in 2011 in the U.S. (1). Similarly, U.S. euthanasia rates declined from ~2.6 million in 2011 to 1.5 million in 2019.

Australia was chosen as a comparison for this study for its similar improvements in LRRs during the same time period (2011–2019), and for its even greater reduction in euthanasia rates. In Australia, the RSPCA is the largest provider of animal shelter care and reported an intake of only 85,033 dogs and cats in 2018–2019 (4), compared to 107,900 in 2011–2012. The result of a 21.2% decrease (5). In 2018–2019, dog euthanasia rates were 12.72% and cats of 22.94% (4), down from 25.6% for dogs and 47.1% for cats in 2011–2012 (5). This was largely credited to a decline in dog intake numbers, from 55,563 in 2011–2012 (5), to 33,863 in 2018–2019, though cat intakes in RSPCA shelters hovered above 50,000 annually (4).

Previous research suggests that these improvements occurred due to human education (6, 7), spay and neuter programs (8), and the transferring of non-human animals from shelters to rescue organizations that create more space and capacity to assist companion animals (9, 10). Other potential factors include improved data collection and sharing between the animal shelter and welfare groups (11), holding off-site adoption events (12), and a general improvement in positive attitudes toward non-human species in society over the past 40 plus years (13–15). In this paper, we contend that professionalizing shelter management, engaging with non-profit social enterprise activities that generate self-sustaining revenue by selling goods or services (e.g., retail operations, selling data software, etc.), and improving the efficiency of daily operations has contributed to more positive human perceptions of companion animals, decreased animal caregiver burnout, and improved LRRs at animal shelters. We first review the literature related to changing human perceptions of non-human animals, profiling animal shelter workers and caregiver burnout, and non-profit social enterprises related to animal shelters before describing our methods and findings from a cross-country comparison study of two large animal shelters, one from Australia and the other from the U.S.

## Literature Review

### Changing Human Perceptions of Non-human Animals

Human-animal studies have focused on the discontinuity of both humans and non-human animals in society, where discourse has shifted to concentrate on animals as mutually beneficial components for both human and non-human societies (16–21). Contemporary human-animal studies are most broadly understood as the examination of interactions among human and non-human animals, emphasizing the expression of non-human agency (22–24). In her seminal book, “*Animals in Society: An Introduction to Human-Animal Studies*,” DeMello (25) considers the conceptual construct of non-human animals within the Euro-American cultural context and the ways it reinforces and perpetuates hierarchical human relationships. Govindrajan (23) utilizes a multispecies ethnographic framework to deconstruct the entanglement of various theoretical concepts (e.g., interspecies relatedness), suggesting that non-human animals have a profound influence on shaping the relationship with human societies through agency, intention, and emotional capacity.

Applied ethology explores animal behavior and welfare of domesticated animals including companion animals (26). Findings center on positive and negative ramifications of human influences, with a strong argument against such concepts as behaviorism and operant conditioning due to their stresses on anthropomorphic hierarchical assumptions that neglect to view non-human animals as agents that co-create mutually beneficial relationships (27). However, in their study of wildlife-human perspectives in modernized countries, Manfredo et al. (15) contend that anthropomorphizing non-human animals, or seeing non-human animals as more human-like, has contributed to pro-non-human animal societal shifts due to power-based domination perspectives giving way to mutualism orientations, “Individuals with strong mutualism orientations would consider wildlife as part of their broader social community, deserving of rights and caring treatment” (p. 2).

### Profiling, Animal Shelter Workers, and Caregiver Burnout

In an attempt to better understand adoption and euthanasia rates, multiple studies examined the potential variables causing certain dogs and cats to be adopted over others. Hill and Murphy (28) employed linear regression models to determine that “dog size, personality, behavior, and level of obedience training” contributed to individual adoption success. A similar study focusing on the length of stay at two “no-kill” animal shelters in New York, showed a direct correlation between the age of the animal and time spent at the facility, where positive outcomes are more likely for puppies than other animals (29). Leonard (30) conducted an ethnographic study at the Washington Humane Society on the cultural bias toward animals based on color associations and literary traditions in Western cultures. She explicitly described the role of “Big Black Dog Syndrome” (BBDS) where large black dogs of all breeds have a difficult time being adopted and are typically the most likely to be euthanized. However, Sinski et al. (31) tested BBDS for its efficacy and found that support for BBDS was mostly anecdotal or theoretical. Data from their study did not support BBDS theory but some shelters still implement strategies such as “applying brightly-colored collars, bows, or bandannas to dark-coated animals” (p. 640), which may suggest positive upticks in adoption rates by making dogs appear more attractive to humans.

Euthanasia affects animal shelter employees and volunteers, leading to burnout and turnover that further increases the costs associated with animal rescue efforts. Anderson et al. (32) surveyed 54 shelter managers across the U.S. where an average of 869 dogs and cats per shelter were euthanized each year. Shelter managers cited emotions of sadness, crying, anger, and depression, which contributed to a 74% employee burnout rate, and 24% turnover rate. “These findings confirm that performing euthanasia can have serious and problematic ramifications for shelter staff and shelter operations” (p 569). Workers are also at risk of emotional or cognitive distress due to often limited resources (e.g., financial, medicinal, staff) that may hinder the quality of care (33).

### Non-profit Social Enterprises

In the U.S., the animal shelter and rescue industry spend an estimated $3.3 to $3.5 billion U.S. dollars annually (34, 35). Research has been conducted in business and management studies on certain aspects of the global consumer pet and veterinarian industries [see McEachern and Cheetham (36), Song and Lim (37), Lemke et al. (38), and Muldowney (39)], but few studies have explored the animal shelter industry from a (non-profit) social entrepreneurship lens. This is perhaps surprising for such a large industry with deeply embedded cultural, emotional, and social ties (40). Globally, non-profits and non-governmental (NGOs) charitable organizations are increasingly affected by the reduction of private donations and government funding, as well as increasing competition from evolving market forces (33). In the U.K. alone, estimates conclude that non-profit funding was cut by $2.8B from 2010 to 2016, deeming it the “Great Recession” for charitable organizations (41–43).

In response, non-profits and NGOs are beginning to reshape the way they conduct business. Ko and Liu (43) emphasize three domains a non-profit can engage to become more financially sustainable and transform into a social enterprise: (1) enact commercial revenue streams, (2) create a professional organizational form, and (3) legitimize a social-commercial business model. Social entrepreneurship (SE) refers to “using social innovations that leverage entrepreneurship to create social value, in a sustainable and market-oriented triple-bottom-line approach” [(44), p. 202], that focuses on social, economic, and environmental justice. Social enterprises are predominantly identified within the non-profit sector [see Fowler (45), Taylor et al. (46), Anderson and Dees (47), Dees et al. (48), Pomerantz (49), and Nicholls (50)]. Non-profit animal shelters, like universities, often rely on government funding, donations, grants, and nominal (adoption) fees to persist. However, most universities operate as non-profit social enterprises by expanding beyond tuition and fee revenue to sell dining packages, merchandise, tickets to sporting events, housing, etc. Non-profit animal shelters must add revenue-generating activities where they sell a good or service such as operating a café to be considered a non-profit social enterprise.

“Mainstream” SE theory frames the literature toward innovative hybrid business models that consider mission-oriented organizations within a market-based dichotomy [see Alter (51)]. Austin and Seitanidi (52) emphasize cross-sector collaboration that strengthens long-term strategic alliances for non-profit social enterprises (NSEs). Bull and Ridley-Duff (53) contend that ethics should be emphasized in SE and proffer a rules-based framework to moral and political choices for entrepreneurs regarding decisions of economic exchange, legal form, and social value orientation. Non-profit SEs that are adapting to new methods of funding must not only consider what to commodify, but how that commodification ripples throughout society within associative, cooperative, and responsible forms of business (53). While Alters' (51) typology of social enterprises provides practitioners a method to analyze multiple models for market exchange and scalability, non-profit social enterprise animal shelters must also consider the non-human animals in their care.

The issue that then arises is how to commodify, professionalize, and account for organizational change activities within animal shelters without sacrificing the stated mission, or the rights, agency, and welfare of non-human animals (51, 54–56). In the complementary field of wildlife ecotourism (for its intersection of human livelihoods and non-human welfare), Thomsen et al. (57) contend that a multispecies livelihoods approach may help to balance human socioeconomic stressors with non-human animal welfare. They take a post-humanist approach to define multispecies livelihoods as “the right for human and non-human animal species to not only exist but to secure the necessities of life in a manner that does not infringe on another species' right to live except for sustenance hunting or legitimate safety concerns to foster optimal conditions for wildlife-human coexistence” (p. 4). We argue here that non-profit animal shelters can transform into social enterprises to remain financially solvent while staying true to their stated mission of rescuing and caring for non-human animals and improving LRRs.

## Methods

This comparative, qualitative study investigated how a large U.S. animal shelter and a similar-sized Australian animal shelter made significant improvements in their LRRs since 2011, and questioned how their relative successes could be replicated, if at all, and under what conditions. This study leveraged an inductive critical philosophical assumption that employed a bottom-up approach, imperative to understand the context that actors face (58). The study was conducted in an Intermountain West, U.S. city, where local governments contract out the largest non-profit animal shelter to assist with efforts in animal rescue, fight cruelty and abuse, run prison dog training programs, as well as manage daily operations to take care of homeless companion animals (59). They receive an average of 41 new animals daily totaling nearly 15,000 dogs and cats per year, in an area with a human population of fewer than 1,000,000 people. The humane society in Australia is also considered to be the largest in its state and handled more than 56,000 animals in the fiscal year 2017–2018, in an area with an estimated population of more than 6,000,000 people. From here forward, the U.S. Intermountain West humane society will be referred to as the U.S. Animal Shelter, and the Australian based one will be referred to as the Australian Animal Shelter.

### Data Collection

Research was conducted in Australia by the first two authors, and in the U.S. by the first three authors. The two shelters were selected for comparison based on their similarities in terms of the shelter's size and inclusion of social enterprise activities. Other shelters could have been selected, but logistical access to conduct the research was also a factor to make the study feasible for the researchers. Documents (online and internal publications shared with the authors) provided comparative insight. Over a 2-week period, 10 semi-structured interviews and brief participant observations were completed in Australia. Over the following 6 months, 11 interviews, along with participant observation (volunteering, job shadowing), took place in the US. For example, participant observation included the researchers volunteering on a marketing campaign for a major fundraising event in the U.S., observing volunteer coordinators as they performed daily tasks, cleaning out kennels and walking dogs while speaking with staff and volunteers, observing surgery, and assisting with light administrative duties. Though similar activities were conducted during the limited time in Australia, the researchers assisted the U.S. shelter two to three times per week over the 6 months. Field notes were handwritten at the end of each day, which provided the researchers an opportunity to reflect on past observations and perceptions during and after the study to help analyze the context of working in a shelter environment.

Each of the 21 formal interviewees were full-time paid employees, part of the administrative staff, and ranged in responsibility from volunteer coordinator to executive director. More than 30 additional informal interviews took place while volunteering and conducting participant observation with workers and volunteers at the shelters. Collective demographic information of the formal interviewees included age ranges from 27-64, where 15 were female and 6 were male. Nineteen had at least a bachelor's degree, 10 had a graduate degree, and all had been with their respective organizations between 2 and 12 years.

### Data Analysis

All participants were anonymized to protect identities and foster candid responses. All interviews were digitally recorded and transcribed to ensure accuracy. All authors coded, categorized, and analyzed responses thematically resulting in three key themes, with saturation reached prior to the conclusion of interviews. The Central Queensland University Ethics Committee approved this study (Application Number 0000020941).

## Findings and Discussion

Three key themes emerged from the interviews and are presented in Table 1 below. These themes include: (1) Professionalizing shelter management, (2) Engaging with non-profit social enterprise activities, and (3) Improving the efficiency of daily operations.

**Table 1:** Overview of three key themes and related sub-themes.

**Professionalizing shelter management**	**Engaging with non-profit social enterprise activities**	**Improving the efficiency of daily operations**
**Key Themes**		
• Shelter employee tasks and goals were standardized, and performance standards were enhanced • Employees behave more professionally, improved professional communication, and built trust with stakeholders • Volunteer coordinators were hired to streamline in-kind and monetary donations • High performing employees were retained, and new employees had higher education and or more business experience	• Modern facilities and innovative space-use were integral to positive engagement and perceptions • Diversifying revenue streams was critical to financial sustainability • Retail operations provided a better customer experience resulting in better adoption rates and funds generated • Funds generated through ancillary revenue streams improved positive perceptions of the shelters	• Adaptive management focused on the professional development of staff and creation of leadership roles • Successful workplace culture linked goals of organization to performance • Innovation generated self-sustaining revenue streams to support non-profit efforts • Executive pay was tied to mission-oriented outcomes rather than cumulative donations

### Key Findings Theme #1: Professionalizing Shelter Management

The Australian and U.S. Animal Shelters transformed their management approach to become more professional and efficient in daily operations. They embraced a non-profit social enterprise model to expand revenue streams such as selling products that included animal shelter tracking software, and pet food and supplies through retail stores, to not solely rely on fluctuating donations and grants. This was accomplished through targeted internal measures such as raising the quality of work expectations for current employees and targeting well-qualified hirees, with external measures of engaging stakeholders through community involvement activities, maintaining an active online social media presence, and building relationships. Other key changes included shifting executive and full-time staff compensation incentives from funds raised to mission-stated outcomes, holding regular employee evaluations, creating development and growth opportunities (personal and advancement) where employees could earn raises and bonuses, and hiring human resource managers with corporate experience to establish clear professional conduct and communication expectations.

In 2011–2012, both shelters proactively reorganized professional standards and expectations amongst current employees. Volunteers were previously managed by another volunteer, and there was little structure or oversight regarding volunteers' work, schedules, and expertise. Volunteer coordinators were hired into paid administrative roles to streamline volunteer operations, hold volunteers accountable, and generate increased in-kind and monetary donations. Each shelter developed a small fee training session for first-time volunteers in order to improve the quality of work and establish buy-in. Once a volunteer reached ~80–100 h of time donated, they would be recruited to participate on more complicated projects such as working with shy dogs, training other volunteers, or helping to coordinate targeted high-donor outreach. The volunteer coordinators tracked more than 1,000 volunteers' hours and used these data to demonstrate improved outcomes to large donors, resulting in increased amounts and frequency. Volunteers who “stood out” were recognized in monthly newsletters, and even led to job opportunities for some. It no longer became acceptable to operate under conditions where a passion for animals solely drove decision making. At each shelter, human resource officers and executive leadership emphasized professional working criteria that included: opportunities for leadership positions, a safe work environment, accountability, performance reviews, and pathways for employees to build a career within the organization. Respondent #1 depicted the more efficient approach to hiring:

We've got a talent management approach. So we try to invest in our best people. We profile the workforce around fit into different areas. Whether you're a high performer, a mediocre performer, poor performer, and you're treated in different ways based on how you slot into that organizational workforce profile. We invest in our really good people. I would say that there's no way you would have been able to bring a high-quality applicant into what we had previously. I just don't think they would have stuck around. But it's putting in place new leadership abilities, capabilities, and creating a work environment where high potential successful people can come and build a career with the organization, where previously, that wasn't the case.

Respondent #1 also shared that by increasing professional standards and opportunities for advancement, they were able to hire candidates with higher education and or more experience. Though this resulted in slightly higher salaries, they were able to meet market-based salaries by creating additional revenue streams. Employees hired expressed gratitude that they were able to earn a livable wage while also being passionate about helping animals. This fostered a culture of employee satisfaction where multiple respondents felt empowered and valued in their roles.

Clear measures around engagement and leadership, variable pay, talent management approaches to human resources, maximal workforce output, and investments in a competitive talent pool reverberated within each organization for operational effectiveness. For example, leadership roles were created to invest in volunteers that saved an estimated ~$2.5M in labor per year at the Australian Animal Shelter. Programs, databases, and targeted events were all shown to improve performance, which ensured support via donations and awareness. These activities included: improved volunteer onboarding training; paired volunteer mentoring where a volunteer with 80–100 plus h trained new volunteers; volunteer hours, expertise, and demographics were tracked at the individual level in excel databases that facilitated consistency on different annual projects or events as the same volunteer worked on the same project; and volunteers could be selected for different events such as gala fundraisers or community “5-km runs” based on the volunteer's interests and expertise. This also led to new organizational partnerships as active volunteers felt appreciated and expressed a desire to “do more,” and facilitated discussions between local companies and the shelters that led to major sponsorships and donations. These activities also helped to improve organizational culture, as Respondent #3 articulated:

One of the biggest problems was between volunteers and staff because of the inattentiveness of our volunteer coordinator at the time. If they [volunteers] had problems, they were going directly to the executive director. That scared staff away. The thinking or the advice was don't talk to volunteers. After the change, we targeted anybody that volunteered more than 80 h and invited them to a meeting to develop volunteer mentors and those groups helped develop our programs.

Organizational strategies were reevaluated, and it was determined that the shelters had to operate more like for-profit businesses to increase productivity, revenue, and trust. The U.S. Animal Shelter also reorganized in a similar fashion. Both shelters stabilized their previously rapid turnover within a couple of years with the hiring of well-educated and experienced employees.

The shelters raised public awareness of their improved shelter management through enhancing their online presence, resulting in public perceptions becoming more positive. Respondent #16 stated:

We are professional, and we're transparent. And that is how you will keep the trust of other organizations and other businesses that might want to work with you, and also the public.

The shelters began communicating directly with potential adopters through improved social media activity and engagement such as prompt responses during business hours and posting more frequently, and increased adoption rates by providing professional pictures and biographical information about the adoptable pets online. Once at the shelter, the adoption interview and processing paperwork were streamlined to facilitate an easier, more retail-like adoption experience. The shelters also credit their professional transformation for building trust with stakeholders that resulted in improved LRRs and helped each shelter raise between $12 and 14 million U.S. dollars to build new retail-style adoption centers.

### Key Findings Theme #2: Engaging With Non-profit Social Enterprise Activities

When the first two authors went to conduct interviews and participant observation at the Australian Animal Shelter, they had to emotionally prepare themselves as they expected to visit what they thought was commonplace shelter infrastructure of animals in cages, invoking images of a prison setting. Though they had heard about “best practices” and newer animal shelters emerging across the animal shelter industry, they had never personally experienced it. The first two authors volunteered in three shelters and visited another 15 over the previous 10 years in the South and Intermountain West regions of the U.S. After taking a tour of the facilities the first author reflected:

I expected to see heart-wrenching dogs in concrete cages, barking incessantly and the look of fear in their eyes, but what we were met with was surprising, to say the least. While we waited for our contact, we were directed to the onsite retail cafe where felines were allowed to roam free and interact with guests while enjoying a beverage or snack in a very comfortable setting. After grabbing coffee, we were led to the back of the building and the differences between my expectations and reality became glaringly apparent. There were no poorly painted concrete walls or gray floors. No large rooms segmented by chain-link dividers. I saw no grungy dogs laying on the concrete or a poor excuse for a mat. We were exposed to something different.

The dogs were placed in brightly painted large rooms with all-glass walls, and each was outfitted with a nice bed, clean water, and a toy or two. The next thing that became obvious was how many employees interacted inside the rooms with the dogs. The shelter had a rotating schedule of volunteers and employees that would not only walk the dogs at set intervals but would come in and play or provide company for the dogs, and each dog had been recently bathed and brushed. Though this is a “best practice” in many shelters, the frequency and more than 4 h of daily enrichment activities astounded the researchers.

The waiting area for adoptions was nicely decorated and split into two sections. Adoptions were finalized on one side with easy access to veterinarians on the other. The final portion of the tour ended in a warehouse-style retail shop with everything a new or experienced dog parent could imagine, similar to a U.S. PetSmart, and at fair prices. Clear signs were posted throughout, indicating that profits from the store would be directly funneled back to support the shelter, providing consumers a sense of “doing even more good.” The shelter had several convenient retail locations throughout the local community.

After touring the facilities, the second author described the experience as:

It was a breath of fresh air-literally and metaphorically. From the very beginning, we were set at ease by watching the constant interactions with the animals housed at the shelter. Once in the holding area, we weren't overwhelmed by the common smell of urine and disinfectant. Everything was clean, the dogs appeared calmer and more excited rather than scared. The relaxing environment also led to the appearance of decreased fear-based behaviors in the dogs. There was less barking and cowering in corners or under beds.

Volunteers shared that this made it easier to predict the dog's behavior and how the prospective adopter may perceive the animal. This seemed to set the stage for a positive experience for the human without the excessive stress of seeing animals in more common cage-like settings. Respondent #7 shared:

When I started, the average was like 2 weeks to find a home and then there would be those few dogs that we would get more concerned about because they would be here for months… I don't see that anymore. Now it's 4–7 days when they are on the adoption floor to find a home… and we just don't see these dogs that are here for months, and to be fair, very few cats are here for so long either.

In addition to the enhanced human and non-human shelter experience and retail operations, the Australian Animal Shelter invested in other revenue streams. These included shelter software, microchipping, thrift shops, crematoriums, puppy parties, and renting space to generate more stable income beyond traditional fundraising, grant writing, and philanthropic donations. The U.S. Animal Shelter was about to break ground on a similar retail-oriented shelter at the time of research and has since built similar operations.

### Key Findings Theme #3: Improving Efficiency of Daily Operations

Improvements in daily operations can be attributed to three primary foci that are shaped by underlying factors of organizational change management: strategy, innovation(s), and workplace culture. They were driven by core competencies, nimble business philosophies, (iterative) process improvement, performance metrics, alignment, communications, relationship building, and leadership goals. Respondent #2 shared, “my role [as director] is obviously to make sure that board policy, overall, is introduced and followed, the mission is sustainable, legislative stuff as far as workplace health and safety, and particularly the mission to save lives.”

In both shelters, significant emphasis was placed on adaptive management that integrates professional development and emotional capacities (often) associated with non-profit organizations (e.g., passion, intention, values). Respondent #5 represented this well:

Traditionally, a lot of welfare groups have taken on people that turn up at an interview and say, “I love animals.” That's great. We're all here because we love animals, but there's a bit more, too, that we need… We're constantly working with government about policy changes, and we often look to other agencies and welfare organizations' best practices. You can't get that without having people with the experience and background doing it.

A few procedures were intended to be strictly adhered to (e.g., evaluating animals and deciding which to go into adoption), but an organization's willingness and capability to adopt new practices are vital to resilience for social enterprises (60–62). Implementing innovative strategies toward non-human animal welfare and agency were key to the success of operations. Respondent #11 explained how animal rights and welfare were intrinsically linked to performance:

We have six key organizational KPIs [key performance indicators]. Two of those are financial. If we hit the financial KPIs it opens up funds, a bonus type arrangement. Essentially, what we've decided to do as an organization is share the success of the organization with those people who determine whether we are going to be successful or not. It's not all about providing bonuses, it's making people more accountable and responsible for turning up and getting the job done.

In addition to tying KPIs such as LRRs to employee performance, other innovative strategies at each shelter included a rebranding of the shelter's image and reputation in the local community. They were able to share improved adoption statistics, the improved living conditions of the animals in their care compared to their previous operations, increased volunteer engagement and appreciation, and an uptick in donations as proof that their efforts were targeted to improve animal welfare. The shift to become a non-profit social enterprise had considerable impacts on the outcomes of each establishment in organizational culture and overall operational efficiencies. These changes emerged throughout several aspects of each shelter, which influenced policy and procedure, technological-systems, (physical) facilities, organizational structure, human resources, and financials.

### Positive Impacts on Human Perceptions, Live Release Rates, and Animal Caregiver Burnout

Clancy and Rowan (63) reviewed the historical record of companion animal demographics in the U.S. They contend that human perceptions of non-human animals have increasingly become more positive since the 1970s, when companion and feral animals were considered “overpopulated” due to a lack of legislation, desexing, and education. The U.S. and Australian Animal shelters previously faced large intake numbers of animals and not enough homes to adopt them out to, as Respondent #4 described:

Twenty-five years ago, it was not so “hip” as it is now to adopt and there wasn't this adoption pride of where you got your animals… Pounds, including our own, [were] really high volume—a lot of euthanasias for time and space, and kind of icky places.

As public interest in animal rights and welfare increased, a cultural shift emerged in the way people view animals. Respondent #19 explained:

Attitudes have changed, you know, people are more likely to sort of see animals as members of their family, which is a transition. I mean, it took a long time for a lot of people to realize, for example, you can't just chain a dog outside.

In response to this cultural shift, cramped, dark, and depressing shelters have been criticized as being inhumane, and pressures to improve the welfare and quality of life for animals within shelters have proliferated, resulting in improved LRRs (64). Innovative facilities were created in many locations, and programs designed to provide behavioral enrichment to the animals became more commonplace. This increased engagement with stakeholders, maximized adoption efficiency, and helped to reduce animal caregiver burnout. Respondent #10 at the Australian Animal Shelter shared that “the major positive changes have been around going from something like 50%, euthanasia, to 11%.” This is a major improvement, but it should be noted that the LRRs are calculated slightly differently in Australia compared to the U.S. as the LRR includes non-health-related euthanasia for humane reasons.

The U.S. shelter also increased LRRs from an average of 83% to above 98%. Respondent #17 shared:

We're quite open about euthanasia. And we will never say that we don't, but we do not euthanize animals for space, and we do not euthanize a rehomeable animal. When we euthanize dogs, if it's not because of health, it's because of behavior issues. And sometimes there's just no addressing them.

The two shelters in this study embraced a positive retail experience approach that improved the human and non-human experience and decreased time in shelter for animals. The positive retail experience approach transcended building aesthetics and offering products for sale. Shelter visitors were made to feel that they were “part of the solution,” and that by adopting or purchasing a retail item they were also contributing to the shelter's mission to help save non-humans' lives. One of the volunteers at the Australian Animal shelter described how the visitors became a part of the “animal community” that stood for positive animal welfare. The first two researchers even noticed the transition in their own speech after conducting this study. The first author shared:

When an acquaintance asks, “what kind of dogs do you have?,” we respond that we have two *rescue* pups. We think that they are mostly border collies, but it doesn't matter. They're perfect for us.

When combined with the professionalization of shelter management and operations, as well as other non-profit social enterprise revenue-generating activities (i.e., retail store, software), an array of benefits emerged. Respondent #20 described that with improved volunteer coordination and a more welcoming environment, they were able to spend more time with the animals:

In terms of live release rate and things like that, these animals out here can go crazy in a penned environment within a very short period of time. We can give each animal 4 h a day of enrichment. So that means getting out, sitting down, training them. My objectives here are to get the animals that don't make it to our adoption pens to an affiliate a rescue Group, a rescue partner, so that by doing that we raise our live release rate, and lower our euthanasia rate.

Several respondents reported that volunteer engagement increased and that both shelters were able to hire animal behavior experts to work with animals who would have previously been euthanized. In most cases, the respondents stated that with extra time and support, dog behavior improved, and it was rare that an individual could not be rehabilitated. At the U.S. shelter, dog adoption rates improved so greatly that they were able to collaborate with other non-profits who flew and trucked dogs in from high-kill shelters in other states to meet local demand for dogs. Every single respondent in the study expressed that morale and animal caregiver burnout had improved. Even though horrific animal cruelty cases persist, they described the general attitude and environment was more positive and hopeful. Respondents also stated that they felt they were making a positive difference for animals in their work.

## Conclusion

The study is limited in its scope due to a relatively small sample size. Though the time for data collection was limited in Australia for logistical purposes, a longer period of participant observation would provide more data. Though the small sample size makes it challenging to generalize broad theoretical or practical approaches, these case studies showed that more research should be conducted on how animal shelters can apply non-profit social enterprise activities in their quotidian practices. Future studies should address these limitations and improve the diversity of the study by focusing on non-urban areas, and non-English (predominantly) speaking countries. The key findings indicated that non-profit animal shelters can, under certain conditions, successfully transition into a social enterprise by professionalizing shelter management, diversifying revenue streams, and enhancing operations. To accomplish this, non-profit animal shelters may need to sell a product or service (e.g., a pet retail store and animal shelter tracking software) and focus on two key factors. First, they must tie their executive compensation to mission-stated outcomes (e.g., live release rates) rather than revenue generation (e.g., large donations), and second, professionalize their daily operations (e.g., organizational structure, professional communication, human resource initiatives, etc.). The three key themes may contribute to alternative pathways for animal shelters to improve LRRs relatively quickly, and sustainably. If non-profit animal shelters can embrace these approaches then they may enhance independent financial solvency, promote multispecies welfare, while staying committed to their stated-missions.

## Data Availability Statement

The raw data supporting the conclusions of this article will be made available by the authors, without undue reservation.

## Ethics Statement

The studies involving human participants were reviewed and approved by The Central Queensland University Ethics Committee. The patients/participants provided their written informed consent to participate in this study.

## Author Contributions

JT and BT collected data at both research sites. KC collected data at USA research site. All authors contributed to the data analysis and write-up.

## Conflict of Interest

The authors declare that the research was conducted in the absence of any commercial or financial relationships that could be construed as a potential conflict of interest.
